# Impact of QTL minor allele frequency on genomic evaluation using real genotype data and simulated phenotypes in Japanese Black cattle

**DOI:** 10.1186/s12863-015-0287-8

**Published:** 2015-11-19

**Authors:** Yoshinobu Uemoto, Shinji Sasaki, Takatoshi Kojima, Yoshikazu Sugimoto, Toshio Watanabe

**Affiliations:** National Livestock Breeding Center, Nishigo, Fukushima 961-8511 Japan; Shirakawa Institute of Animal Genetics, Japan Livestock Technology Association, Nishigo, Fukushima 961-8511 Japan

**Keywords:** BovineHD, Genomic prediction, Heritability estimation, Japanese Black cattle, Minor allele frequency, Simulation study

## Abstract

**Background:**

Genetic variance that is not captured by single nucleotide polymorphisms (SNPs) is due to imperfect linkage disequilibrium (LD) between SNPs and quantitative trait loci (QTLs), and the extent of LD between SNPs and QTLs depends on different minor allele frequencies (MAF) between them. To evaluate the impact of MAF of QTLs on genomic evaluation, we performed a simulation study using real cattle genotype data.

**Methods:**

In total, 1368 Japanese Black cattle and 592,034 SNPs (Illumina BovineHD BeadChip) were used. We simulated phenotypes using real genotypes under different scenarios, varying the MAF categories, QTL heritability, number of QTLs, and distribution of QTL effect. After generating true breeding values and phenotypes, QTL heritability was estimated and the prediction accuracy of genomic estimated breeding value (GEBV) was assessed under different SNP densities, prediction models, and population size by a reference-test validation design.

**Results:**

The extent of LD between SNPs and QTLs in this population was higher in the QTLs with high MAF than in those with low MAF. The effect of MAF of QTLs depended on the genetic architecture, evaluation strategy, and population size in genomic evaluation. In genetic architecture, genomic evaluation was affected by the MAF of QTLs combined with the QTL heritability and the distribution of QTL effect. The number of QTL was not affected on genomic evaluation if the number of QTL was more than 50. In the evaluation strategy, we showed that different SNP densities and prediction models affect the heritability estimation and genomic prediction and that this depends on the MAF of QTLs. In addition, accurate QTL heritability and GEBV were obtained using denser SNP information and the prediction model accounted for the SNPs with low and high MAFs. In population size, a large sample size is needed to increase the accuracy of GEBV.

**Conclusion:**

The MAF of QTL had an impact on heritability estimation and prediction accuracy. Most genetic variance can be captured using denser SNPs and the prediction model accounted for MAF, but a large sample size is needed to increase the accuracy of GEBV under all QTL MAF categories.

**Electronic supplementary material:**

The online version of this article (doi:10.1186/s12863-015-0287-8) contains supplementary material, which is available to authorized users.

## Background

The development of single nucleotide polymorphism (SNP) array technology has enhanced the genetic dissection of complex traits, and this SNP information can be directly utilized in cattle breeding programs using genomic selection [1, 2]. In addition, whole genome sequence (WGS) data are becoming increasingly available for cattle, and WGS data are expected to yield a better understanding of complex traits, which can capture all of the genetic variance and predict an accurate genomic estimated breeding value (GEBV), by accounting for all the variants including quantitative trait loci (QTLs) [3, 4].

A recent report showed that the SNPs significantly associated with a complex trait explain only a fraction of the phenotypic variance in human height, and this has been called the “missing heritability” problem [5]. It has been argued that missing heritability is due to imperfect linkage disequilibrium (LD) between SNPs and QTLs, and the extent of LD between SNPs and QTLs depends on differences in the minor allele frequency (MAF) between SNPs and QTLs [6]. SNPs with similar MAF can potentially have high LD, but SNPs with very different MAF cannot have high LD. In cattle populations, QTLs may have a lower MAF than SNPs on low-density SNP arrays, because these are designed to work in several different breeds. In this case, the genetic variation explained by SNPs will be lower than that due to low LD between SNPs and QTLs with low MAF. Meat from Japanese Black cattle is known to have the unique characteristic of a high degree of marbling; the cattle are genetically distant from other European breeds at the genome level [7]. The extent of LD between SNPs and QTLs in Japanese Black cattle may differ from that in other cattle breeds, and it is necessary to evaluate the impact of MAF of QTLs on the genomic evaluation in this target population.

Heritability estimation and GEBV prediction are measures of goodness-of-fit in reference populations and have predictive ability in test populations, respectively. The amount of genetic variance not captured by SNPs affects the maximum predictive ability [8]. On the other hand, increasing the goodness-of-fit will not necessarily increase the predictive ability, because of the model over-fitting problem [9]. The heritability estimation and prediction accuracy depend on several factors such as the genetic architecture of a trait (e.g., QTL heritability, number of QTLs, and distribution of QTL effect), the evaluation strategy (e.g., SNP marker density and prediction method), and population size [6, 9–12]. Therefore, it is important how heritability estimation and GEBV prediction depends on these factors in different MAF of QTLs.

The objective of this study was to evaluate the impact of MAF of QTLs on heritability estimation and accuracy of GEBV prediction, and how that depends on the genetic architecture (QTL heritability, number of QTLs, and distribution of QTL effect), the evaluation strategy (SNP density and prediction model), and population size. We performed a simulation analysis based on a reference-test validation design, which used real genotype data to account for the extent of LD in Japanese Black cattle.

## Methods

Genotypes for this study were obtained from previously published data [13]. All animal experiments were performed according to the Guidelines for the Care and Use of Laboratory Animals of Shirakawa Institute of Animal Genetics, and this research was approved by Shirakawa Institute of Animal Genetics Committee on Animal Research (H21-2). We have obtained the written agreement from the cattle owners to use the samples.

### Data

In this simulation analysis, real genotype data were used to account for the extent of LD in Japanese Black cattle. Complete descriptions of the experimental population and SNP information were reported previously by Uemoto et al. [13]. Briefly, a total of 1444 Japanese Black cattle, which were 653 steers from two slaughterhouses in Japan [14] and 791 cows from farms managed by a large cooperative farming company in Japan [15], were genotyped using the Illumina BovineHD BeadChip (HD) (Illumina, San Diego, CA, USA), and 593,696 SNPs on autosomal chromosomes assessed by the exclusion criteria of MAF < 0.01, call rate < 0.95, and Hardy–Weinberg equilibrium test < 0.001 were used in this study. To avoid having very close relatives in the data, the animals with large off-diagonal elements in the genomic relationship matrix (GRM) were excluded (a cut-off value of ± 0.4 for off-diagonal elements), and the SNPs were then reassessed by the same criteria. A total of 1368 animals and 592,034 SNPs were then used in the simulation study. These animals were low relatives with the progeny of 438 sires, and the mean, median, and maximum number of progenies per sire were 3.1, 2, and 24, respectively. The distribution of progenies per sire was shown in Additional file 1: Figure S1.

### Simulation design

In this study, we simulated the true breeding value (TBV) and phenotypes under the different scenarios varying the following factors: different MAF categories, QTL heritability, number of QTLs, and distribution of QTL effect. After generating TBV and phenotypes, the QTL heritability was estimated and the prediction accuracy of GEBV was assessed under different conditions varying the following factors: different SNP densities, prediction models, and size of the reference-test populations by a reference-test validation design. The factors considered in the simulation study are summarized in Table 1, and shown in detail below. The impact of the MAF of QTLs on genomic evaluation under different genetic architecture was evaluated in scenarios 1 and 2. In addition, the impact of the MAF of QTLs on genomic evaluation under different evaluation strategy and population size was evaluated in scenarios 3 and 4, respectively.

**Table 1:** Factors for different scenarios in a simulation study

	Scenario			
Factor	1	2	3	4
MAF^a^	All, High, Low	All, High, Low	All, High, Low	All, High, Low
QTL heritability	0.2, 0.4, 0.8	0.4	0.4	0.4
Number of QTLs	500	50, 100, 300, 500, 1000, 2000	500	500
Distribution of QTL effect^b^	EquV	Gamma, EquV	EquV	EquV
SNP density^c^	50 K	50 K	7 K, 50 K, 7K_to_HD, 50 K_to_HD, HD	50 K
Prediction model^d^	Model (1) with G_Y_	Model (1) with G_Y_	Model (1) with G_V,_ G_Y_, and G_S_, Model (2)	Model (1) with G_Y_
Size of reference set	1231	1231	1231	200, 400, 800, 1200
Size of test set	137	137	137	1168, 968, 568, 168

^a^MAF, Minor allele frequency; All, 0.01 ≤ MAF ≤ 0.5; High, 0.05 < MAF ≤ 0.5; Low, 0.01 ≤ MAF ≤ 0.05

^b^Gamma, Gamma distribution model; EquV, Equal variance model

^c^7K, 50 K and HD, Illumina infinium BovineLDv1.1, BovineSNP50v2, and BovineHD BeadChips, respectively; 7 K_to_HD and 50 K_to_HD, Imputations were performed from 7 K and 50 K to HD, respectively

^d^G_V_, VanRaden's G matrix; G_Y_, Yang's G matrix; G_S_, Speed's G matrix

**Fig. 1:**
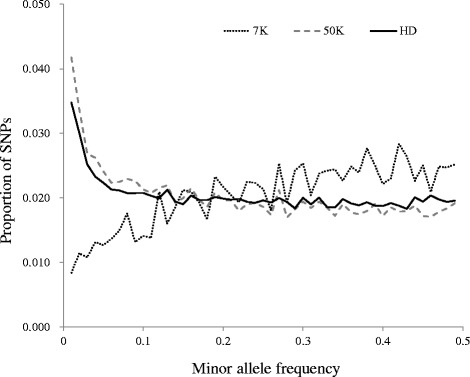
Distribution of minor allele frequencies for SNPs under different SNP densities. The x-axis indicates the MAF of SNPs, and the y-axis represents the proportion of SNPs in each MAF category. 7 K, 50 K, and HD are SNP markers on Illumina infinium BovineLDv1.1, BovineSNP50v2, and BovineHD BeadChips, respectively

In this simulation, 36,478 and 6316 SNPs on the BovineSNP50v2 BeadChip (50 K) and the BovineLDv1.1 BeadChip (7 K) (Illumina, San Diego, CA, USA), respectively, were designated as SNP markers. The distribution density of MAF of SNPs on 7 K, 50 K, and HD is plotted in Fig. 1. The MAF distribution shows a low ratio of SNPs on 7 K and a high ratio of SNPs on 50 K and HD at low MAF. The remaining 555,556 SNPs that are present in the HD but not in the 50 K and 7 K were assumed as candidate QTLs. For SNP density, three types of SNPs were used in this simulation. First, SNPs on 7 K and 50 K were used, and this scenario involved imperfect LD between SNPs and QTLs (and named as the imperfect LD SNPs). Second, the HD genotype was imputed from SNPs on 50 K (50 K_to_HD) and 7 K (7K_to_HD) by the BEAGLE (v4.0) software [16]. We performed a 10-fold cross-validation to have imputed HD genotype in this population, and the detail of imputation was reported previously by Uemoto et al. [13]. The imputed SNPs were then reassessed by the same exclusion criteria as described above, and 585,015 and 588,547 SNPs were used in the 7K_to_HD and 50 K_to_HD, respectively. The detail of the imputation error ratio was shown by Uemoto et al. [13], and the average correlation between true and imputed genotypes were 0.98 in 50 K_to_HD and 0.93 in 7 K_to_HD. This scenario involved some SNPs being QTLs but with a low imputation error ratio (and named as the imputed SNPs). Third, all SNPs on the HD were used as SNPs, and this scenario assumed that WGS data were available and some SNPs were QTLs itself (and named as the perfect LD SNPs).

For candidate QTLs, three MAF categories were defined as follows: a low MAF group (0.01 ≤ MAF ≤ 0.05), a high MAF group (0.05 < MAF ≤ 0.5), and an all MAF group (0.01 ≤ MAF ≤ 0.5). A total of 50, 100, 300, 500, 1000, and 2000 QTLs were randomly selected from candidate QTLs in each MAF group. Hill et al. [17] showed that the distribution of allele frequency affecting additive genetic variance is under the U-shaped distribution and $\mathit{\mathsf{f}}(p)\propto \frac{\mathsf{1}}{p\left(\mathsf{1}-p\right)}$. For the all MAF group, the U-shaped distribution was assumed as the distribution of QTL allele frequency (0.01 ≤ *p* ≤ 0.5), and the ratio of the integrated values for low MAF, ${\displaystyle {\int}_{\mathsf{0.01}}^{\mathsf{0.05}}f(p)dp}$, and high MAF, ${\displaystyle {\int}_{\mathsf{0.05}}^{\mathsf{0.5}}f(p)dp}$, were 0.36 and 0.64, respectively. Therefore, QTLs with low and high MAFs in the all MAF group were randomly selected from the ratio 0.36:0.64, respectively.

We assumed the use of a polygenic model in the simulation, because this is a reasonable assumption for the majority of complex traits in cattle. The phenotype was simulated by summing all true QTL genotypic values and the residual effect, that is, ${\mathit{\mathsf{y}}}_{\mathit{\mathsf{i}}}={\displaystyle \sum_j^m{x}_{ij}{b}_j}+{e}_i$, where *m* is the number of QTLs, *x*_*ij*_ is the genotype for the *j*-th QTL of the *i*-th animal (coded as 0, 1, or 2 for the homozygote, heterozygote, and the other homozygote, respectively), *b*_*j*_ is the allele substitution effect of the *j*-th QTL, and *e*_*i*_ is the residual effect generated from $\mathit{\mathsf{N}}\left(\mathsf{0},\kern0.5em {\sigma}_g^{\mathsf{2}}\left(\mathsf{1}/{h}^{\mathsf{2}}-\mathsf{1}\right)\right)$. ${\displaystyle \sum_j^m{x}_{ij}{b}_j}$ is TBV, ${\sigma}_g^{\mathsf{2}}$ is the total genetic variance of TBV, and *h*^*2*^ is the setting value of QTL heritability. Three setting values of QTL heritability (*h*^*2*^ = 0.20, 0.40, and 0.80) were used to generate phenotypes.

In this study, two different distributions of the QTL effect were assumed. The first model was a gamma distribution model in which the QTL effect was generated from a gamma distribution with a shape parameter of 0.4 and scale parameter of 1.66 [2]. The second model was an equal variance model in which the QTL effect was assumed as ${b}_j=\frac{\mathsf{1}}{\sqrt{\mathsf{2}{p}_j\left(\mathsf{1}-{p}_j\right)}}$, where *p*_*j*_ is MAF of *j*-th QTL. In the equal variance model, the QTL effect was assumed in that all QTLs had contributed to QTL variance equally (Var(*b*_*j*_) = 1 in this assumption) if linkage equilibrium was assumed among QTLs. The signs of QTL effects were randomly selected, and total QTL variance was adjusted to 100 × *h*^*2*^ in both distribution models.

### Statistical analysis

The generated data were analyzed by the genomic best linear unbiased prediction (GBLUP) method with the following model:

1 $$ \mathbf{\mathsf{y}}={\mathbf{1}}_{\mathbf{n}}\mu +\mathbf{\mathsf{X}}\mathbf{\mathsf{u}}+\mathbf{\mathsf{e}} $$

where **y** is the phenotypic values, **1**_**n**_ is a vector of *n* ones, μ is the mean, **X** is the design matrix for random effects, **u** is the additive genetic effect with $\mathbf{\mathsf{u}}\sim \text{\textit{\textsf{N}}}\left(\mathsf{0},\kern0.5em \mathbf{\mathsf{G}}{\sigma}_u^{\mathsf{2}}\right)$, and **e** is the residual effect with $\mathbf{\mathsf{e}}\sim \text{\textit{\textsf{N}}}\left(\mathsf{0},\kern0.5em \mathbf{\mathsf{I}}{\sigma}_e^{\mathsf{2}}\right)$. **G** is a GRM using all SNPs in each SNP density. ${\mathit{\mathsf{\sigma}}}_{\mathit{\mathsf{u}}}^{\mathsf{2}}$ is the additive genetic variance, and ${\sigma}_e^{\mathsf{2}}$ is residual variance. We also used the following model:

2 $$ \mathbf{\mathsf{y}}={\mathbf{1}}_{\mathbf{n}}\mu +\mathbf{\mathsf{X}}{\mathbf{\mathsf{u}}}_{\mathbf{\mathsf{L}}}+\mathbf{\mathsf{X}}{\mathbf{\mathsf{u}}}_{\mathbf{\mathsf{H}}}+\mathbf{\mathsf{e}} $$

where **u**_**L**_ is the additive genetic effect attributed to the low MAF SNPs with ${\mathbf{\mathsf{u}}}_{\mathbf{\mathsf{L}}}\sim \text{\textit{\textsf{N}}}\left(\mathsf{0},\kern0.5em {\mathbf{\mathsf{G}}}_{\mathbf{\mathsf{L}}}{\sigma}_{u_L}^{\mathsf{2}}\right)$, and **u**_**H**_ is the additive genetic effect attributed to the high MAF SNPs with ${\mathbf{\mathsf{u}}}_{\mathbf{\mathsf{H}}}\sim \text{\textit{\textsf{N}}}\left(\mathsf{0},\kern0.5em {\mathbf{\mathsf{G}}}_{\mathbf{\mathsf{H}}}{\sigma}_{u_H}^{\mathsf{2}}\right)$. **G**_**L**_ is a GRM using SNPs with low MAF, and **G**_**H**_ is a GRM using SNPs with high MAF in each SNP density. ${\sigma}_{u_L}^{\mathsf{2}}$ and ${\sigma}_{u_H}^{\mathsf{2}}$ are the additive genetic variances attributed to the SNPs with low and high MAFs, respectively, and ${\sigma}_e^{\mathsf{2}}$ is the residual variance. We defined three different GRMs as follows:

VanRaden’s GRM (**G**_**V**_): The first GRM, **G**_**V**_, was proposed by VanRaden [18] and is calculated as follows:

$$ {\mathbf{\mathsf{G}}}_{\mathbf{\mathsf{V}}}=\frac{\mathbf{\mathsf{Z}}\mathbf{\mathsf{Z}}\mathbf{\hbox{'}}}{\mathsf{2}{\displaystyle \sum_{j=\mathsf{1}}^m{p}_j\left(\mathsf{1}-{p}_j\right)}} $$

where *m* is the number of SNPs, *p*_*j*_ is the frequency of the second allele of *j*-th SNP, and the elements of ***Z*** are calculated as follows:

$$ {\mathit{\mathsf{z}}}_{ij}={\mathit{\mathsf{x}}}_{ij}-\mathsf{2}{p}_j $$

where *x*_*ij*_ is the number of the second allele of the *i*-th individual at the *j*-th SNP.

Yang’s GRM (**G**_**Y**_): The second GRM, **G**_**Y**_, was proposed by Yang et al. [6] and is computed as follows:

$$ {\mathbf{\mathsf{G}}}_{\mathbf{\mathsf{Y}}}=\frac{\overline{\mathbf{\mathsf{Z}}}\;\overline{\mathbf{\mathsf{Z}}}\mathbf{\hbox{'}}}{m} $$

where $\overline{\mathbf{\mathsf{Z}}}$ is the **Z** matrix but with each element scaled based on the allele frequency of each locus as follows:

$$ {\overline{\mathit{\mathsf{z}}}}_{ij}=\frac{z_{ij}}{\sqrt{\mathsf{2}{p}_j\left(\mathsf{1}-{p}_j\right)}} $$

Speed’s GRM (**G**_**S**_): The third GRM, **G**_**S**_, was proposed by Speed et al. [19] and is calculated as follows:

$$ {\mathbf{\mathsf{G}}}_S=\frac{\mathbf{\mathsf{W}}\mathbf{\mathsf{W}}\mathbf{\hbox{'}}}{{\displaystyle \sum_{j=\mathsf{1}}^m{k}_j}} $$

where *k*_*j*_ is the weighting factor of the *j*-th SNP accounted for LD and the elements of **W** are calculated as follows:

$$ {w}_{ij}=\sqrt{k_j}{\overline{z}}_{ij} $$

Speed et al. [19] proposed a method for weighting markers to account for LD. Their method, linkage-disequilibrium adjusted kinships (LDAK), examines the local SNP correlation caused by LD and computes optimal SNP weights by solving a linear program. We calculated the weighting factor *k*_*j*_ and the LD-adjusted GRM (**G**_**S**_) by the LDAK software with default parameters and LD decay function. When analyzing high density SNPs (i.e., imputed SNPs and perfect LD SNPs), the weighting factors were calculated twice as suggested.

After calculating these three GRMs, 0.00001 was added to diagonal elements of each GRM to avoid near singularity problems. We used the three GRMs in model (1) and **G**_**Y**_ in model (2). The QTL heritability ${\mathit{\mathsf{h}}}_{\mathsf{1}}^{\mathsf{2}}$ and ${\mathit{\mathsf{h}}}_{\mathsf{2}}^{\mathsf{2}}$ for model (1) and (2), respectively, are calculated as follows,

$$ {\mathit{\mathsf{h}}}_{\mathsf{1}}^{\mathsf{2}}=\frac{\sigma_u^{\mathsf{2}}}{\sigma_u^{\mathsf{2}}+{\sigma}_e^{\mathsf{2}}} $$

$$ {\mathit{\mathsf{h}}}_{\mathsf{2}}^{\mathsf{2}}=\frac{\sigma_{u_{\mathit{\mathsf{L}}}}^{\mathsf{2}}+{\sigma}_{u_{\mathit{\mathsf{H}}}}^{\mathsf{2}}}{\sigma_{u_{\mathit{\mathsf{L}}}}^{\mathsf{2}}+{\sigma}_{u_{\mathit{\mathsf{H}}}}^{\mathsf{2}}+{\sigma}_e^{\mathsf{2}}} $$

### Validation test of heritability estimation and prediction accuracy

Under each scenario, we replicated a reference-test validation design 300 times. In each reference-test experiment, data were randomly split into two disjointed sets, that is, 137 animals (one-tenth of all animals) in the test population and the remaining 1231 animals in the reference population. In each replica, this approach was performed only one time. In addition, to evaluate the impact of MAF of QTLs under different population size, 200, 400, 800, and 1200 animals were randomly selected as the reference population, and the remaining 1168, 968, 568, and 168 animals were used as the test population, respectively. Phenotypes of animals in the test population were masked in each replicate, and we estimated QTL heritability in the reference population and predicted the GEBV in the test population using the ASREML 3.0 program [20]. After predicting the GEBV, the prediction accuracy was assessed using Pearson’s correlation between TBV and GEBV in each test population of the validation set. The mean and standard deviation (SD) of 300 replicates was then calculated.

## Results

### Extent of LD between SNPs and QTLs

**Fig. 2:**
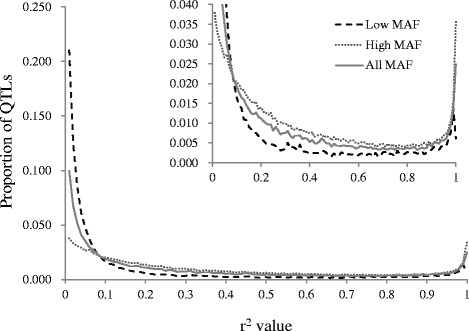
Proportion of linkage disequilibrium value (r^2^) between QTLs and adjacent SNPs. The plot on the right upper corner is the zoomed area of the bigger plot. The x-axis indicates the r^2^ value between QTLs and SNPs, and the y-axis represents the proportion of QTLs in each minor allele frequency (MAF) category (All, Low, and High). The r^2^ values between QTLs and both adjacent SNPs were calculated, and then the maximum value of r^2^ between two QTL-SNP intervals was chosen to plot in each QTL. The parameters used were the same as those under scenario 1

Under all scenarios, three MAF categories were defined to evaluate the impact of MAF of QTLs. To evaluate the impact of MAF of QTLs on the extent of LD between SNPs and QTLs, the extent of LD between SNPs on 50 K and QTLs in each MAF category is shown in Fig. 2. The extent of LD between SNPs and QTLs was evaluated using the r^2^ value, which is a measure of LD. The r^2^ values between QTLs and both adjacent SNPs were calculated by PLINK software [21]. The maximum value of r^2^ between two QTL-SNP intervals was chosen in each QTL, and the density distributions of r^2^ for three MAF categories were then plotted. The parameters used were the same as those used in scenario 1. In this result, most QTLs with low MAF had a lower r^2^ value than those with high MAF. The r^2^ value of QTLs with all MAF was between that of QTLs with low and high MAFs. The mean values of r^2^ for all, high, and low MAFs were 0.294, 0.360, and 0.184, respectively. This shows that the extent of LD between SNPs and QTLs is higher in the QTLs with high MAF than that in those with low MAF.

### The genetic architecture

**Fig. 3:**
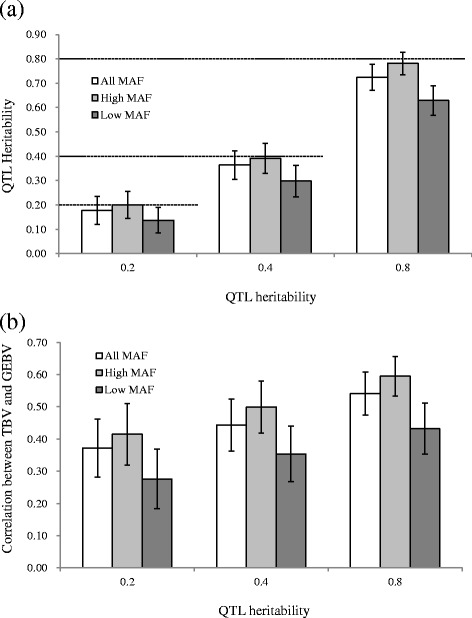
Results obtained from scenario 1. Estimated QTL heritability and correlation between true breeding and genomic estimated breeding values are calculated. The x-axis indicates the true QTL heritability, and the y-axis represents mean values of 300 replicates for the estimated QTL heritability (**a**) and the correlation between true breeding value (TBV) and genomic estimated breeding value (GEBV) (**b**). The results of varying minor allele frequency (MAF) categories (All, Low, and High) and QTL heritabilities (0.20, 0.40, and 0.80) are shown. The whiskers represent the standard deviation of 300 replicates

We evaluated the impact of MAF of QTLs on genomic evaluation under different QTL heritability in scenario 1, and the estimated QTL heritability and correlation between TBV and GEBV are shown in Fig. 3. The estimated QTL heritability was close to the setting value and a higher correlation was observed as the QTL heritability was increased in each MAF category. For the MAF of QTLs, the estimated QTL heritability and correlation between TBV and GEBV for QTLs with high MAF has the highest value, and the values of all MAF were between those of low and high MAFs in each setting value of QTL heritability. In addition, as the setting value was increased from 0.20 to 0.80, the differences in the results between high and low MAFs increased in QTL heritability (from 0.06 to 0.15, respectively) and correlation between TBV and GEBV (from 0.14 to 0.16, respectively).

**Fig. 4:**
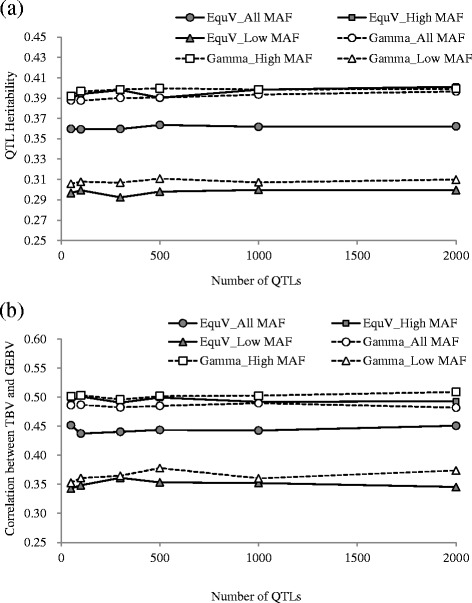
Results obtained from scenario 2. Estimated QTL heritability and correlation between true breeding and genomic estimated breeding values are calculated. The x-axis indicates the number of QTLs, and the y-axis represents mean values of 300 replicates for the estimated QTL heritability (**a**) and the correlation between true breeding value (TBV) and genomic estimated breeding value (GEBV) (**b**). The results of varying minor allele frequency (MAF) categories (All, Low, and High), number of QTLs (50, 100, 300, 500, 1000, and 2000), and distribution of QTL allele substitution effect (Gamma, gamma distribution model; EquV, equal variance model) are shown

We evaluated the impact of MAF of QTLs on genomic evaluation under different number of QTLs and distribution of the QTL effect in scenario 2, and the estimated QTL heritability and correlation between TBV and GEBV are shown in Fig. 4. For QTL number, the estimated QTL heritability and correlation remained constant, regardless of the number of QTLs in each MAF category.

For the distribution of QTL effect, the results of the QTLs with high and low MAFs followed a similar trend between the two distribution models, whereas different results were observed between two distribution models in the QTLs with all MAFs. The results of high and all MAFs showed similar trends in the gamma distribution model, and the estimated QTL heritability and correlation between TBV and GEBV were about 0.39 and 0.50, respectively. On the other hand, the results of all MAFs were lower than those of high MAF in the equal variance model, and the values of estimated QTL heritability and correlation between TBV and GEBV were about 0.36 and 0.44 for all MAF and 0.39 and 0.50 for high MAF, respectively.

### The evaluation strategy

We evaluated the impact of the MAF of QTLs on genomic evaluation under different evaluation strategy for SNP density and prediction model in scenario 3. Goodness-of-fit was measured by the Akaike information criterion (AIC) to compare the prediction models. The AIC is defined as $\mathit{\mathsf{A}}\mathit{\mathsf{I}}\mathit{\mathsf{C}}=\mathsf{2}\mathit{\mathsf{v}}-\mathsf{2} \ln \left(\mathit{\mathsf{likelihood}}\right)$, where *v* is the number of variance components. This formula shows that the goodness of fit is high, if the AIC is low. The estimated QTL heritability, AIC, and correlation between TBV and GEBV are shown in Table 2, Table 3, and Table 4, respectively.

**Table 2:** Heritability estimation in scenario 3

		All MAF^a^		High MAF^a^		Low MAF^a^	
SNP^b^	Prediction model^c^	Mean	SD	Mean	SD	Mean	SD
7 K	Model (1) with G_V_	0.28	0.05	0.32	0.05	0.20	0.06
	Model (1) with G_Y_	0.30	0.05	0.33	0.05	0.23	0.06
	Model (1) with G_S_	0.30	0.05	0.33	0.05	0.24	0.06
	Model (2)	0.30	0.05	0.33	0.05	0.23	0.06
50 K	Model (1) with G_V_	0.33	0.06	0.38	0.06	0.24	0.06
	Model (1) with G_Y_	0.36	0.06	0.39	0.06	0.30	0.06
	Model (1) with G_S_	0.38	0.06	0.40	0.06	0.34	0.07
	Model (2)	0.37	0.06	0.39	0.06	0.34	0.06
7K_to_HD	Model (1) with G_V_	0.34	0.06	0.39	0.06	0.24	0.06
	Model (1) with G_Y_	0.37	0.06	0.40	0.06	0.30	0.06
	Model (1) with G_S_	0.41	0.07	0.41	0.07	0.39	0.07
	Model (2)	0.39	0.06	0.40	0.06	0.38	0.06
50K_to_HD	Model (1) with G_V_	0.34	0.06	0.39	0.06	0.25	0.06
	Model (1) with G_Y_	0.37	0.06	0.41	0.06	0.30	0.07
	Model (1) with G_S_	0.41	0.07	0.42	0.07	0.40	0.07
	Model (2)	0.40	0.06	0.40	0.06	0.40	0.06
HD	Model (1) with G_V_	0.35	0.06	0.39	0.06	0.25	0.06
	Model (1) with G_Y_	0.38	0.06	0.41	0.06	0.31	0.07
	Model (1) with G_S_	0.42	0.07	0.41	0.07	0.40	0.07
	Model (2)	0.40	0.06	0.40	0.06	0.41	0.06

^a^MAF, Minor allele frequency; All MAF, 0.01 ≤ MAF ≤ 0.5; High MAF, 0.05 < MAF ≤ 0.5; Low MAF, 0.01 ≤ MAF ≤ 0.05

^b^7K, 50 K and HD, Illumina infinium BovineLDv1.1, BovineSNP50v2, and BovineHD BeadChips, respectively; 7 K_to_HD and 50 K_to_HD, Imputations were performed from 7 K and 50 K to HD, respectively

^c^G_V_, VanRaden's genome relationship matrix (GRM); G_Y_, Yang's GRM; G_S_, Speed's GRM

**Table 3:** Model fitness measured by Akaike information criterion (AIC) in scenario 3

		All MAF^a^		High MAF^a^		Low MAF^a^	
SNP^b^	Prediction model^c^	Mean	SD	Mean	SD	Mean	SD
7 K	Model (1) with G_V_	6164	63	6145	66	6191	61
	Model (1) with G_Y_	6162	63	6145	66	6188	61
	Model (1) with G_S_	6162	63	6146	66	6187	61
	Model (2)	6163	63	6147	66	6186	61
50 K	Model (1) with G_V_	6159	63	6139	65	6188	62
	Model (1) with G_Y_	6155	63	6139	65	6181	62
	Model (1) with G_S_	6155	63	6142	65	6175	62
	Model (2)	6155	63	6140	65	6163	62
7K_to_HD	Model (1) with G_V_	6158	63	6138	65	6189	62
	Model (1) with G_Y_	6155	63	6138	65	6182	62
	Model (1) with G_S_	6156	63	6147	65	6171	62
	Model (2)	6154	63	6139	65	6155	62
50K_to_HD	Model (1) with G_V_	6157	63	6137	65	6188	62
	Model (1) with G_Y_	6154	63	6137	65	6181	62
	Model (1) with G_S_	6155	63	6146	65	6169	62
	Model (2)	6153	63	6138	65	6152	62
HD	Model (1) with G_V_	6157	63	6136	65	6188	62
	Model (1) with G_Y_	6154	63	6137	65	6180	62
	Model (1) with G_S_	6155	63	6147	65	6168	62
	Model (2)	6152	63	6138	65	6150	62

^a^MAF, Minor allele frequency; All MAF, 0.01 ≤ MAF ≤ 0.5; High MAF, 0.05 < MAF ≤ 0.5; Low MAF, 0.01 ≤ MAF ≤ 0.05

^b^7K, 50 K and HD, Illumina infinium BovineLDv1.1, BovineSNP50v2, and BovineHD BeadChips, respectively; 7 K_to_HD and 50 K_to_HD, Imputations were performed from 7 K and 50 K to HD, respectively

^c^G_V_, VanRaden's genome relationship matrix (GRM); G_Y_, Yang's GRM; G_S_, Speed's GRM

**Table 4:** Correlation between true breeding value and genomic breeding value in scenario 3

		All MAF^a^		High MAF^a^		Low MAF^a^	
SNP^b^	Prediction model^c^	Mean	SD	Mean	SD	Mean	SD
7 K	Model (1) with G_V_	0.41	0.08	0.48	0.08	0.30	0.09
	Model (1) with G_Y_	0.42	0.08	0.48	0.08	0.32	0.09
	Model (1) with G_S_	0.42	0.08	0.48	0.08	0.33	0.09
	Model (2)	0.42	0.08	0.48	0.08	0.33	0.09
50 K	Model (1) with G_V_	0.43	0.08	0.50	0.08	0.32	0.09
	Model (1) with G_Y_	0.44	0.08	0.50	0.08	0.35	0.09
	Model (1) with G_S_	0.44	0.08	0.49	0.08	0.37	0.09
	Model (2)	0.44	0.08	0.50	0.08	0.41	0.09
7K_to_HD	Model (1) with G_V_	0.44	0.08	0.50	0.08	0.32	0.09
	Model (1) with G_Y_	0.45	0.08	0.50	0.08	0.35	0.09
	Model (1) with G_S_	0.44	0.08	0.48	0.08	0.38	0.08
	Model (2)	0.45	0.08	0.50	0.08	0.44	0.08
50K_to_HD	Model (1) with G_V_	0.44	0.08	0.51	0.08	0.32	0.09
	Model (1) with G_Y_	0.45	0.08	0.51	0.08	0.36	0.09
	Model (1) with G_S_	0.44	0.08	0.48	0.08	0.39	0.08
	Model (2)	0.46	0.08	0.51	0.08	0.46	0.08
HD	Model (1) with G_V_	0.44	0.08	0.51	0.08	0.32	0.09
	Model (1) with G_Y_	0.45	0.08	0.51	0.08	0.36	0.08
	Model (1) with G_S_	0.44	0.08	0.48	0.08	0.39	0.08
	Model (2)	0.46	0.08	0.51	0.08	0.47	0.08

^a^MAF, Minor allele frequency; All MAF, 0.01 ≤ MAF ≤ 0.5; High MAF, 0.05 < MAF ≤ 0.5; Low MAF, 0.01 ≤ MAF ≤ 0.05

^b^7K, 50 K and HD, Illumina infinium BovineLDv1.1, BovineSNP50v2, and BovineHD BeadChips, respectively; 7 K_to_HD and 50 K_to_HD, Imputations were performed from 7 K and 50 K to HD, respectively

^c^G_V_, VanRaden's genome relationship matrix (GRM); G_Y_, Yang's GRM; G_S_, Speed's GRM

Differences in the SNP density have an impact on heritability estimation and GEBV prediction. For model (1) with G_Y_, the results of 50 K were higher than those of 7 K in all MAF categories. For example, from the QTLs with all MAFs, the results of 50 K and 7 K were 0.36 and 0.30 for QTL heritability and 0.44 and 0.42 for correlation between TBV and GEBV, respectively. The results of imputed SNPs (i.e., 7 K_to_HD and 50 K_to_HD) were higher than those of 7 K and 50 K, and were very close to the results of perfect LD SNPs (i.e., HD) in all MAF categories. For example, from the QTLs with all MAFs, the results of both 50 K_to_HD and 7 K_to_HD were 0.37 for QTL heritability and 0.45 for correlation between TBV and GEBV, and the results of HD were 0.38 for QTL heritability and 0.45 for correlation between TBV and GEBV. These results indicate that heritability estimation and GEBV prediction depend on the SNP density. However, the different results among SNP densities in each MAF category depend on the prediction model.

For the prediction model, the result of model (1) with G_V_ was similar to that with G_Y_ in the QTL with high MAF, but the difference between the results obtained from G_V_ and G_Y_ increased in the QTL with low MAF. For example, the differences between G_V_ and G_Y_ in the AIC and correlation between TBV and GEBV with 50 K were 0 and 0.00 in the QTL with high MAF but 7 and 0.03 in the QTL with low MAF, respectively. The result of model (1) with G_S_ was similar to or better than that with G_Y_ in the QTL with all and low MAFs, but performed worse in the QTL with high MAF. In particular, the difference in the results between G_S_ and G_Y_ in the QTL with high MAF was increased at larger SNP density. For example, the difference between G_S_ and G_Y_ in AIC and correlation between TBV and GEBV were 1 and 0.00 in 7 K but 10 and 0.03 in HD. In addition, the results of G_S_ with HD in high MAF were 6147 in AIC and 0.48 in the correlation between TBV and GEBV, which represented the worst of all results by other models under the high MAF scenario. The results of model (2) were similar to or better than those of the other three models under all MAF categories. In particular, the results of model (2) with HD in low MAF, which were 6150 in AIC and 0.47 in correlation between TBV and GEBV, representing the best values in the low MAF results.

### Population size

**Fig. 5:**
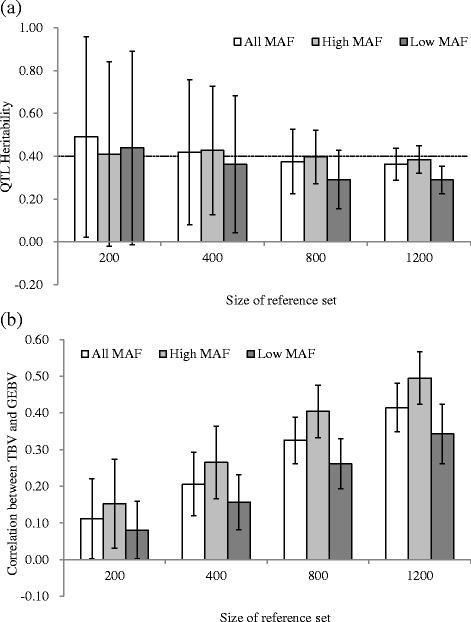
Results obtained from scenario 3. Estimated QTL heritability and correlation between true breeding and genomic estimated breeding values are calculated. The x-axis indicates the size of the reference set, and the y-axis represents mean values of 300 replicates for the estimated QTL heritability (**a**) and the correlation between true breeding value (TBV) and genomic estimated breeding value (GEBV) (**b**). The results of varying minor allele frequency (MAF) categories (All, Low, and High) and size of the reference set (200, 400, 800, and 1200) are shown. The whiskers represent the standard deviation of 300 replicates

In this simulation, the impact of the MAF of QTLs on genomic evaluation under different population size was evaluated in scenario 4. The estimated QTL heritability and correlation between TBV and GEBV are shown in Fig. 5. The results of heritability estimation and GEBV prediction followed a different trend. The mean values of estimated QTL heritability were close to the setting value (0.40) and were almost the same as those among different population sizes, but the SD of the estimated results decreased as the size of the population increased (e.g., from 0.47 to 0.07 in reference size from 200 to 1200, respectively, for all MAFs). The following trend of the results, the mean values of high MAF > all MAF > low MAF, was shown for QTL heritability, when the size of reference set was more than 800. These results indicated that the heritability estimates at lower population sizes are less precise than those at higher population sizes, even if the estimated value is close to the setting value. In addition, the impact of the MAF of QTLs was shown at larger population sizes.

In the GEBV prediction, the correlations between TBV and GEBV were increased as the size of the reference increased (e.g., 0.11–0.41 at reference size 200–1200, respectively, for all MAFs). QTLs with high MAF had the highest value, and the values of all MAFs were between those with low and high MAFs in all reference sizes (e.g., 0.34, 0.41, and 0.50 for low, all, and high MAFs in reference size 1200, respectively). In addition, as the size of the reference increased from 200 to 1200, the difference between the high and low MAFs for the correlations between TBV and GEBV increased from 0.07 to 0.15, respectively.

## Discussion

### The genetic architecture

The differences in the QTL heritability and the distribution of QTL effect had an impact on heritability estimation and GEBV prediction under different MAF categories, but the differences in the number of QTL did not have. The results of the correlation between TBV and GEBV for the number of QTL were the same as those described by Daetwyler et al. [10], because the accuracy of GBLUP is constant regardless of the number of QTLs. The trend of the results for QTL heritability was similar to that described by Yang et al. [6].

For the distribution of QTL effect, the genetic variance of the *j*-th QTL is theoretically calculated as $\mathsf{2}{\mathit{\mathsf{p}}}_{\mathit{\mathsf{j}}}\left(\mathsf{1}-{\mathit{\mathsf{p}}}_{\mathit{\mathsf{j}}}\right){\alpha}_{\mathit{\mathsf{j}}}^{\mathsf{2}}$, where *p*_*j*_ is the allele frequency of QTLs and *α*_*j*_ is the QTL effect [22]. This formula shows that the QTL effect will increase as the allele frequency decreases, if the genetic variance is constant. Therefore, the QTLs with low MAF must have a higher QTL effect than those with high MAF to contribute to the total genetic variance. In a real data analysis, findings from a meta-analysis of human height showed that the QTLs with high MAF had small phenotypic effects, whereas the QTLs with low MAF had large effects on this trait such as a function of the MAF [23]. Therefore, missing heritability has focused on the possible contribution of QTLs with low MAF, and the QTLs with low MAF could have an intermediate effect [24]. In this study, the factor for the distribution of QTL effect was evaluated to account for the low-MAF QTL with intermediate effect. In the gamma distribution model, the low-MAF QTL with intermediate effect cannot be defined in the QTL with all MAFs, because the QTL effect was randomly allocated to the MAF. On the other hand, this QTL can be defined in the equal variance model. As an example, the mean values for the QTL effect and QTL variance for the QTL with all MAFs as a function of MAF are shown in Additional file 1: Figure S2. Additional file 1: Figure S2 is drawn from a result of the randomly selected replica under scenario 2 with the parameters for the number of QTLs (500). In this result, no relationship between MAF, QTL effect, and QTL variance was observed in the gamma distribution model, whereas the QTL with low MAF had a higher QTL effect and all QTLs had equal genetic variance in the equal variance model. Therefore, the results of the QTLs with all and high MAFs in Fig. 4 showed the same as that under the gamma distribution model, because of the low contribution of the QTLs with low MAF on the total genetic variance. This result was the similar trend as described by Wientjes et al. [25]. This result also shows that the equal variance model accounts for missing heritability in a simulation when the QTLs are composed of variants with low and high MAFs. If QTLs with a large effect do exist, they are at a low frequency and individually explain a small proportion of genetic variance [26]. Therefore, the equal variance model was used to evaluate the impact of MAF of QTL in all scenarios.

### The evaluation strategy

In this study, three types of SNPs were assumed: the imperfect LD SNPs (7 K and 50 K), the imputed SNPs (7 K_to_HD and 50 K_to_HD), and the perfect LD SNPs (HD). Recently, WGS data are becoming increasingly available for use in cattle, and the 1000 bull genomes project provides annotated sequence variants and genotypes of key ancestor bulls [3]. One of the major advantages of WGS data is that they provide complete information on all the variants of an individual, which include many of the SNPs with low MAF that are not covered by the SNP array. Most of the low MAF variants are only accessible through WGS data, and this information could be important for genomic evaluation. WGS data can be obtained directly by next-generation sequencing techniques or indirectly by genotype imputation. When using imputed SNPs, the impact of imputation error on genomic evaluation must be investigated in genotype imputation. Therefore, the imputed SNPs (indirect information) and the perfect LD SNPs (direct information) were used to evaluate the effectiveness of using WGS data directly or indirectly. The results showed that differences in the SNP density have an impact on heritability estimation and GEBV prediction, especially in the low MAF scenario. For the imperfect LD SNPs, the distribution of MAF for 7 K followed a different trend compared to HD. On the other hand, the distribution of MAF for 50 K had the different values but followed a similar trend compared to that for HD, especially at high MAF. Usually, all classes of MAFs are equally represented on a low density SNP array, while the low MAF class is overrepresented in the WGS data [27]. The difference in MAF distribution between QTL and SNPs indicates the difficulty of capturing genetic variance. Therefore, the results of 7 K were lower than those of 50 K. For the imputed SNPs and the complete LD SNPs, these results were higher than those with 7 K and 50 K in heritability estimation and GEBV prediction. The results of imputed SNPs were very close to those of the complete LD SNPs, even if the imputed SNPs were not in perfect LD with the QTL. The number of missing genotypes affects the accuracy, and the difference in imputation accuracy is larger at low MAFs [13]. However, our results showed that there was little difference in the results between 7K_to_HD and 50 K_to_HD under the low MAF scenario. A previous study reported that the accuracy of GEBV plateaus on increasing the number of SNPs [12]. On the other hand, GEBV prediction can achieve moderately high prediction accuracy under perfect LD between SNPs and QTLs in distantly related human data [28]. Therefore, using the SNPs related to QTLs directly or indirectly is effective for performing heritability estimation and GEBV prediction.

In this study, we showed that the differences of the result among SNP densities in each MAF category depend on the prediction model. For model (1) with G_V_ and G_Y_, the difference of the results between G_V_ and G_Y_ was increased in the QTL with low MAF. Meuwissen et al. [29] suggested that weighted GRM by MAF would have a better result than unweighted GRM, when a high proportion of loci with low MAF are used. G_Y_ is corrected for variance of the allele frequency of each SNP, and gives weight to alleles with low MAF. On the other hand, G_V_ is corrected for the average frequency of heterozygotes, and gives less weight to alleles with low MAF. Therefore, the approach of G_Y_ was better than that of G_V_, especially under the low MAF scenario. For the model (1) with G_S_ reflecting the degree of LD, the difference of the results between G_S_ and G_Y_ in the QTL with high MAF was increased at larger SNP densities, and the result using HD was the worse than that by other prediction models under the high MAF scenario. Lee et al. [30] reported that G_S_ generates biased heritability estimates through the use of denser SNPs, because of too much weight being attributed to the low MAF SNPs. This method accounts for the different extents of LD among SNPs, and weighted SNPs depend on the MAF distribution of SNPs. The distribution of MAF is different between the dense and sparse SNP data, because the proportion of SNPs with low MAF increased as SNP density increased. The low MAF SNPs could be under low LD among SNPs and the proportion of weighted SNPs with low MAF will increase. In this simulation, the proportion of SNPs before and after weighting for G_S_ is different for sparse and dense SNP data, and the proportion of weighted SNPs with low MAF was higher than that with high MAF in imputed SNPs and perfect LD SNPs (Additional file 2: Table S1). Therefore, the result of the QTL with high MAF was the lowest, because many of the low MAF SNPs were weighted in G_S_. To account for the degree of LD between SNPs at larger SNP densities, the haplotype model (such as Sun et al. [31]) could have significant average in low MAF QTL.

For model (2), the results were similar to or better than those from the other three models under all MAF categories, and represented the best value when a higher proportion of QTLs that had low MAF and SNPs on HD were used. This model means that QTL heritability is partitioned by the MAF of SNPs to provide insight into genetic architecture. Some studies have showed that different MAF categories could deliver estimates with little bias and high goodness-of-fit in human [30, 32] and chicken population [33, 34], because high LD is only possible between SNPs with similar MAFs. In addition, Lee et al. [30] also showed that the different MAF categories generate heritability estimates with higher goodness-of-fit in dense SNP data compared with sparse SNP data, especially when a higher proportion of QTLs have low MAF. In principle, fitting more MAF categories in a prediction model could more accurately represent the genetic architecture, but it brings the disadvantage of estimating more parameters. Therefore, Lee et al. [30] used five bins with MAF boundaries 0.1, 0.2, 0.3, 0.4, and 0.5. In this study, two MAF categories (high and low) were fitted in the model (2), because the QTL can be roughly classified into two types: an intermediate effect at low MAF and a small effect at high MAF under missing heritability. Therefore, more MAF categories were not fitted at high MAF. Our results using model (2) with HD were better than those based on five MAF categories in all MAF categories of this simulation (Additional file 2: Table S2). Therefore, model (2) is a robust method in all MAF categories and could capture more of the genetic variance under the low MAF scenario if the WGS data are available.

### Population size

In this simulation, a high proportion of genetic variance was captured in the high MAF scenario. However, our results did not reflect high precision, and the accuracy of GEBV remained lower than that of the simulation study [2], even if the perfect LD SNP was used under the high MAF scenario. Under this simulation, the maximum correlation between TBV and GEBV for the QTLs with all, high, and low MAFs was 0.46, 0.51, and 0.47, respectively, for QTL heritability of 0.40. The main reason for low prediction accuracy could be the size of the reference population. The accuracy of GEBV depends on the size of the reference, and a large sample of animals is needed in the reference population if accurate GEBV prediction is desired [1]. Daetwyler et al. [35] also described the accuracy of GEBV deterministically as follows:

$$ \mathit{\mathsf{r}}=\sqrt{\frac{\mathit{\mathsf{N}}{\mathit{\mathsf{h}}}^{\mathsf{2}}}{\mathit{\mathsf{N}}{\mathit{\mathsf{h}}}^{\mathsf{2}}+\mathit{\mathsf{q}}}} $$

where *N* is the number of individuals in the reference population, *h*^*2*^ is heritability, and *q* is the number of independent loci affecting a trait. This formula shows that the accuracy of GEBV depends on the size of the reference and the number of QTLs. Daetwyler et al. [35] also showed that GBLUP has a constant accuracy for a given *N* and *h*^*2*^, regardless of *q*. Therefore, the accuracy of GEBV increases with a larger reference size.

In this study, the accuracy increased as the size of the reference increased in all MAF categories, and the accuracy did not plateau at the maximum size of the reference (Fig. 5). Under this simulation, there were insufficient DNA samples to evaluate the impact of a larger sample size. Therefore, further study is needed to evaluate the impact of population size on genomic prediction under different MAF scenarios.

### Simulation based on real data

Populations containing related individuals (e.g., cattle) are expected to yield high LD between SNPs and QTLs than populations containing unrelated individuals (e.g., human), because of decreasing effective population size. The LD in cattle follows a similar pattern to that of humans at short distances, but is much larger than that of humans at long distances [1]. The extent of LD between SNPs and QTLs depends on a population structure, and the impact of MAF of QTL must be evaluated by the use of datasets accounting for the extent of LD in a target population. The effective population size of Japanese Black cattle has been reduced because of the intensive use of a few sires with high marbling [36], and the extent of LD could be higher than that in other cattle breeds. There has been little assessment of the MAF of QTLs that affect heritability estimation and GEBV prediction using real cattle data except for Wientjes et al. [25] in Dairy cattle population. Therefore, we used real genotype data in the simulation study, which accounted for the extent of LD in Japanese Black cattle. For the extent of LD in this target population, the extent of LD between SNPs and QTLs increased as the MAF of QTL increased in the scenario with 50 K. The main reason was that 87 % of SNPs in 50 K were high MAF (see Additional file 2: Table S1). On the other hand, only 13 % of SNPs were low MAF, and most of QTLs with low MAF were in low LD with SNPs in 50 K. This indicates that the concordance of MAF between QTL and SNP shows higher LD between two loci in this population.

Under this simulation, we showed that the results of these predicted models with different SNP densities depend on the genetic architecture of objective traits, especially the MAF of QTLs. Recently, some studies have investigated the proportion of genetic variance captured by SNPs and the prediction accuracy of GEBV in small Japanese Black cattle populations using the GBLUP method with G_V_ and 50 K [37–39]. In those studies, a higher proportion of genetic variance was captured for carcass traits, and these proportions were close to the genetic variance previously reported by estimations based on pedigree information. In our results with the scenario of all MAF category and setting QTL heritability (0.40), the genome heritability was 0.33 for the scenario with G_V_ and 50 K, whereas there was genome heritability of 0.40 for the scenario with model (2) and HD. In addition, previous studies show that the some proportion of the genetic variance are not captured by SNPs with high MAF in Holstein cattle population [40, 41] and in chicken population [33]. Therefore, the explanation of a high proportion may be that there are not only the QTLs with high MAF but also strong relationship structures in these populations, because our population was excluded very close relatives to obtain low relationship structure. The strong relationship structures also lead to capture more of genetic variance by SNPs with high MAF. To evaluate the genetic architecture of complex traits, we suggest that it is effective to compare among these prediction models with different SNP densities. However, it is not sufficient for the evaluation of predictive ability, because of the difficulty of obtaining TBV. It may not reflect the predictive ability, even if a higher proportion of genetic variance is captured. In this population, the extent of LD was still higher than that in other beef breeds [13], even if we excluded very close relatives to evaluate the minimum value of QTL heritability and correlation in Japanese Black cattle. These results show that a higher proportion of genetic variance was captured under the high MAF scenario. However, the accuracy of GEBV remained low, and the goodness-of-fit did not increase the prediction accuracy. Therefore, further study including additional animals in the reference population is needed to increase the prediction accuracy.

## Conclusions

The current study evaluated the impact of MAF of QTL on genomic evaluation in a simulation study by assuming different MAFs of QTLs and several factors in Japanese Black cattle. The extent of LD between SNPs and QTLs was higher in the QTLs with high MAF than in those with low MAF. The MAF of QTLs had an impact on heritability estimation and prediction accuracy and that depended on the genetic architecture, evaluation strategy and population size. The genetic architecture results showed that genomic evaluation was affected by the MAF of QTLs combined with the QTL heritability and the distribution of QTL effect. The number of QTL was not affected on genomic evaluation if the number of QTL was more than 50. For the evaluation strategy, different SNP densities and prediction models affected the heritability estimation and genomic prediction, and these depended on the MAF of QTLs. The genetic dissection of complex traits would be possible by comparing the results of these predicted models with different SNP densities. In addition, accurate QTL heritability and GEBV were obtained by using denser SNP information and model (2) under all MAF categories. However, it may not reflect the predictive ability, and a larger sample size is needed to increase the accuracy of GEBV.

### Availability of supporting data

The data sets supporting the results of this article are included within the article and its additional file.

## Additional files

Additional file 1: Figure S1. Distribution of progenies per sire in this population. The x-axis indicates the number of progenies per sire, and the y-axis represents the number of sires. **Figure S2.** QTL effect and QTL variance as a function of minor allele frequency (MAF). The x-axis indicates the MAF of SNPs, and the y-axis represents the QTL effect (a) and QTL variance (b) in a randomly selected replica. The results of varying distributions of QTL allele substitution effects (Gamma, gamma distribution model; EquV, equal variance model) for all MAF, QTL heritability (0.40), and the number of QTLs (500) are shown. (PPTX 51 kb) Additional file 2: Table S1. Number of SNPs before and after weighting for Speed's genomic relationship matrix. **Table S2.** Comparison of two and five minor allele frequency (MAF) categories. (DOC 62 kb)

## Abbreviations

AIC: Akaike information criterion
GBLUP: Genomic best linear unbiased prediction
GEBV: Genomic estimated breeding value
GRM: Genomic relationship matrix
LD: Linkage disequilibrium
MAF: Minor allele frequency
QTL: Quantitative trait locus
SNP: Single nucleotide polymorphism
TBV: True breeding value
WGS: Whole genome sequence
