# Supplementary material for: Impact of QTL minor allele frequency on genomic evaluation using real genotype data and simulated phenotypes in Japanese Black cattle
Source: BMC Genet. 2015 Nov 19;16:134. doi: 10.1186/s12863-015-0287-8 (PMC4653875; doi:10.1186/s12863-015-0287-8)
Supplement: Additional file 1: Figure S1. — Distribution of progenies per sire in this population. The x-axis indicates the number of progenies per sire, and the y-axis represents the number of sires. Figure S2. QTL effect and QTL variance as a function of minor allele frequency (MAF). The x-axis indicates the MAF of SNPs, and the y-axis represents the QTL effect (a) and QTL variance (b) in a randomly selected replica. The results of varying distributions of QTL allele substitution effects (Gamma, gamma distribution model; EquV, equal variance model) for all MAF, QTL heritability (0.40), and the number of QTLs (500) are shown. (PPTX 51 kb) [file 12863_2015_287_MOESM1_ESM.pptx]

## Slide 1
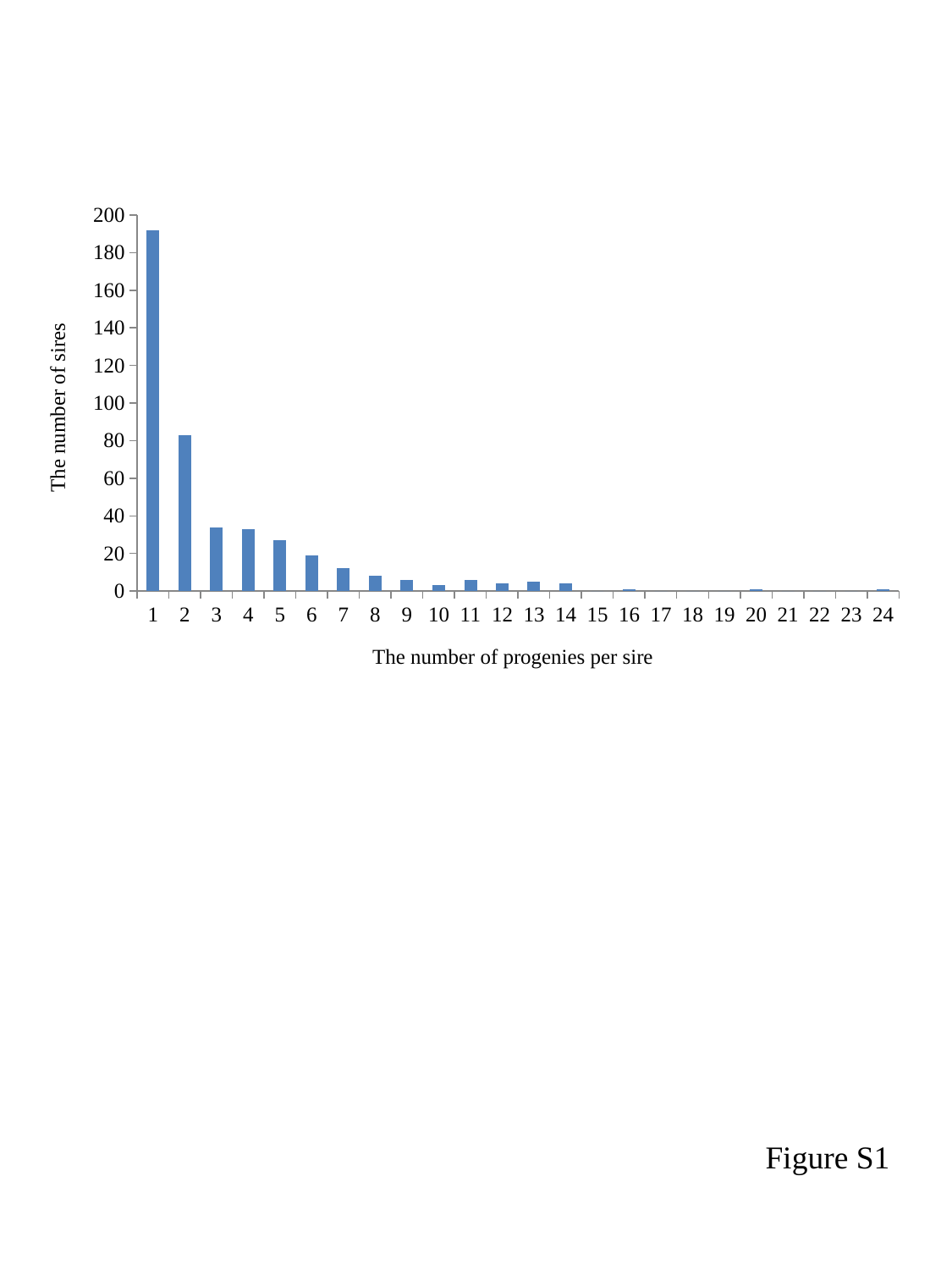

### Chart
| Category | |
|---|---|
| 1 | 192.0 |
| 2 | 83.0 |
| 3 | 34.0 |
| 4 | 33.0 |
| 5 | 27.0 |
| 6 | 19.0 |
| 7 | 12.0 |
| 8 | 8.0 |
| 9 | 6.0 |
| 10 | 3.0 |
| 11 | 6.0 |
| 12 | 4.0 |
| 13 | 5.0 |
| 14 | 4.0 |
| 15 | 0.0 |
| 16 | 1.0 |
| 17 | 0.0 |
| 18 | 0.0 |
| 19 | 0.0 |
| 20 | 1.0 |
| 21 | 0.0 |
| 22 | 0.0 |
| 23 | 0.0 |
| 24 | 1.0 |The number of sires
The number of progenies per sire
Figure S1

## Slide 2
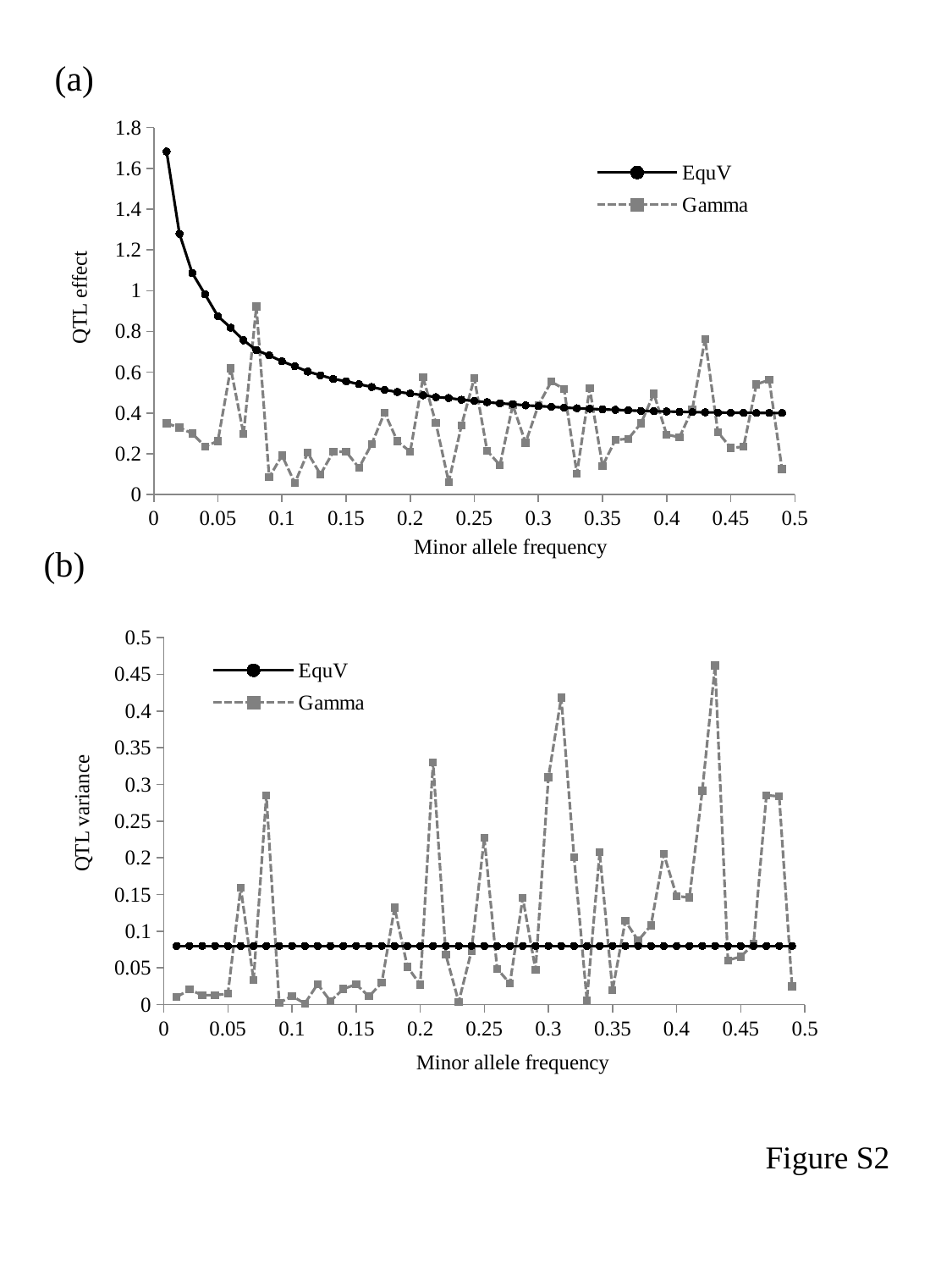

(a)
### Chart
| Category | EquV | Gamma |
|---|---|---|QTL effect
Minor allele frequency
(b)
### Chart
| Category | EquV | Gamma |
|---|---|---|QTL variance
Minor allele frequency
Figure S2
