# Supplementary material for: Impact of QTL minor allele frequency on genomic evaluation using real genotype data and simulated phenotypes in Japanese Black cattle
Source: BMC Genet. 2015 Nov 19;16:134. doi: 10.1186/s12863-015-0287-8 (PMC4653875; doi:10.1186/s12863-015-0287-8)
Supplement: Additional file 2: Table S1. — Number of SNPs before and after weighting for Speed's genomic relationship matrix. Table S2. Comparison of two and five minor allele frequency (MAF) categories. (DOC 62 kb) [file 12863_2015_287_MOESM2_ESM.doc]

Table S1. Number of SNPs before and after weighting for Speed's genomic relationship matrix.

|  |  | Number of SNP^1^ | | | | |
| --- | --- | --- | --- | --- | --- | --- |
| MAF^2^ | Weighting | 7K | 50K | 7K_to_HD | 50K_to_HD | HD |
| Low (0.01-0.05) | Before | 276 | 4690 | 67295 | 66673 | 67101 |
|  | After | 276 | 4624 | 31446 | 31172 | 32553 |
| High (0.05-0.50) | Before | 6040 | 31788 | 517720 | 521874 | 524933 |
|  | After | 6026 | 29163 | 97708 | 97504 | 99363 |
| Ratio (High/Low) | Before | 21.9 | 6.8 | 7.7 | 7.8 | 7.8 |
|  | After | 21.8 | 6.3 | 3.1 | 3.1 | 3.1 |

^1^7K, 50K and HD, Illumina infinium BovineLDv1.1, BovineSNP50v2, and BovineHD BeadChips, respectively.

^2^Minor allele frequency.

Table S2. Comparison of two and five minor allele frequency (MAF) categories.

|  |  | QTL heritability | |  | AIC^2^ | |  | Correlation | |
| --- | --- | --- | --- | --- | --- | --- | --- | --- | --- |
| MAF^1^ | Model | Mean | SD |  | Mean | SD |  | Mean | SD |
| All | Model (2) | 0.40 | 0.06 |  | 6152 | 63 |  | 0.46 | 0.08 |
|  | Model with five MAF categories | 0.40 | 0.06 |  | 6157 | 63 |  | 0.45 | 0.08 |
| High | Model (2) | 0.40 | 0.06 |  | 6138 | 65 |  | 0.51 | 0.08 |
|  | Model with five MAF categories | 0.41 | 0.06 |  | 6141 | 65 |  | 0.51 | 0.08 |
| Low | Model (2) | 0.41 | 0.06 |  | 6150 | 62 |  | 0.47 | 0.08 |
|  | Model with five MAF categories | 0.40 | 0.06 |  | 6164 | 62 |  | 0.44 | 0.08 |

^1^All MAF, 0.01≤MAF≤0.5; High MAF, 0.05<MAF≤0.5; Low MAF, 0.01≤MAF≤0.05.

^2^AIC, Akaike information criterion.
